# Rhizobial Chemoattractants, the Taste and Preferences of Legume Symbionts

**DOI:** 10.3389/fpls.2021.686465

**Published:** 2021-05-04

**Authors:** K. Karl Compton, Birgit E. Scharf

**Affiliations:** Department of Biological Sciences, Life Sciences I, Virginia Tech, Blacksburg, VA, United States

**Keywords:** bacterial survival, flagellar motility, plant-host exudate, plant-microbe signaling, rhizosphere, symbiosis

## Abstract

The development of host-microbe interactions between legumes and their cognate rhizobia requires localization of the bacteria to productive sites of initiation on the plant roots. This end is achieved by the motility apparatus that propels the bacterium and the chemotaxis system that guides it. Motility and chemotaxis aid rhizobia in their competitiveness for space, resources, and nodulation opportunities. Here, we examine studies on chemotaxis of three major model rhizobia, namely *Sinorhizobium meliloti*, *Rhizobium leguminosarum*, and *Bradyrhizobium japonicum*, cataloging their range of attractant molecules and correlating this in the context of root and seed exudate compositions. Current research areas will be summarized, gaps in knowledge discussed, and future directions described.

## Introduction

The endosymbiosis between leguminous plants and rhizobia benefits both parties whereby the bacteria, sheltered and supplied nutrients from the plant, fix nitrogen into ammonia for their host. Several rhizobium-legume combinations have stood out as the model systems for this symbiosis, namely *Sinorhizobium* (*Ensifer*) *meliloti* – *Medicago truncatula*, *Bradyrhizobium japonicum* – *Glycine max*, and *Rhizobium leguminosarum*, the latter able to nodulate clovers, pea, common bean, or others depending on the biovar. These organisms have been used to build our knowledge on the genetics, biochemistry, development, and ecology of the many facets of this interaction. The initiation step of the symbiosis occurs at the tips of young root hairs, which curl and pinch in on a population of rhizobia, allowing access inside the plant cells. Prior to this, the rhizobia must localize themselves to the root hairs and outcomplete other bacteria for this niche (Ames et al., 1980; Ames and Bergman, 1981; Pinochet et al., 1993). This is achieved with chemotaxis and motility, the phenomenon by which bacteria move up a gradient of attractant or down a gradient of repellent. Attractant and repellent signals are many and diverse, as bacteria can respond to carbon sources, heavy metals, osmolytes, pH, light, and temperature (Tso and Adler, 1974; Hazelbauer, 1975; Croxen et al., 2006; Jekely, 2009; Paulick et al., 2017; Webb et al., 2017a). The typical mechanism of chemotactic sensing starts with transmembrane sensor proteins called methyl-accepting chemotaxis proteins (MCPs or receptors), which sense multiple and highly different signals such as single molecules, chemical classes, and physical stimuli (Paulick et al., 2017). MCP-ligand binding modulates the autokinase activity of the internal chemotaxis protein CheA. Phosphorylated CheA transfers its phosphoryl group to the response regulator CheY, which interacts with the flagellar motor to affect a change in its rotation. This two-component system thus controls the movement of the bacterium toward an attractant or away from a repellent by sensing increasing or decreasing ligand binding (Parkinson et al., 2015; Salah Ud-Din and Roujeinikova, 2017).

To study chemotaxis, numerous assays have been used to quantify bacterial behavior, but Adler’s capillary assay remains the gold standard (Adler, 1966, 1973). In short, the method involves filling glass capillaries with a putative attractant solution and placing the capillary into a suspension of bacteria. During incubation, the attractant solution forms a gradient which the bacteria follow inside the capillary. The result is typically measured with colony counts of the capillary contents. A reference capillary containing only buffer is included to account for diffusion of cells, acting as an internal negative control. Chemotaxis values are either reported by subtracting reference counts from test counts or as a coefficient with test counts being divided by the reference. Unfortunately, variations in growth conditions, technical procedures, and bacterial strains make comparing studies between lab groups difficult. For example, one study might define a few thousand cells above background as significant, while another might require >10^5^ cells as a significant response (Aguilar et al., 1988; Barbour et al., 1991; Kape et al., 1991; Meier et al., 2007; Webb et al., 2014).

Here, we review studies that used the capillary assay to derive information about the attractants of *B. japonicum*, *R. leguminosarum*, and *S. meliloti* (Table 1). Other methods such as swim plates are available, but do not offer comparable resolution and so will be excluded (Sampedro et al., 2015). A discussion will focus on chemical classes that have been tested for chemotaxis and their prevalence in plant exudates.

**Table 1:** A catalog of the attractants identified in quantitative chemotaxis assays.

Species	Strain	Attractants found	Weak/not attracants	References
*Sinorhizobium meliloti*	MVII-1	All amino acids	Sugars	Götz et al. (1982)
	Ve 26	Some amino acids such as aspartate, lysine; gluconate	Hydrophobic amino acids; sugars	Burg et al. (1982)
	RCR2011 (SU47)	Luteolin		Caetanoanollés et al. (1988)^∗^
	L5.30	Certain amino acids; sugars, especially sucrose	Glutamine; xylose	Malek (1989)
	2011	4',7-Dihydroxyflavone; 4',7-dihydroxyflavanone; 4,4'-dihydroxy-2'-methoxychalcone; luteolin		Dharmatilake and Bauer (1992)^∗^
	RU11/001	Non-acidic amino acids; monocarboxylates; quaternary ammonium compounds	Aspartate, glutamate; butyrate, formate; flavonoids	Webb et al. (2017a,b), Compton et al. (2018, 2020)
*Rhizobium leguminosarum* biovar. *Phaseoli*	RP8002	Raffinose, sucrose, xylose; apigenin, luteolin, p-hydroxybenzoic acid, 3,4-dihydroxybenzoic acid	Glucose, maltose; vanillyl alcohol; naringenin	Aguilar et al. (1988)
	WU163	L-arabinose, cellobiose, D-glucose, D-ribose, D-xylose	Sucrose; trehalose	Bowra and Dilworth (1981)
*Rhizobium leguminosarum* biovar. *Viciae*	N5	Amino acids; organic acids; sugars such as arabinose, maltose, glucose, xylose	Fucose, sucrose, trehalose	Gaworzewska and Carlile, 1982
	8,401	Small amino acids; sucrose, mannitol, maltose; succinate; apigenin, naringenin, kaempferol	Galactose, ribose; propionate; hesperitin	Armitage et al., 1988
*Bradyrhizobium japonicum*	110spc4	Hydroxycinnamic acids; succinate	Coniferyl alcohol, chlorogenic acid, coumestrol, daidzein, genistein	Kape et al., 1991
	USDA 110	Aspartate, glutamate; organic acids	Non-acidic amino acids; citrate; daidzein, genistein, luteolin; sugars	Barbour et al. (1991)
	10 K	Alanine, glutamate, phenylalanine, threonine; arabinose, mannitol; citrate	Other amino acids; organic acids; sugars	Chuiko et al., 2002
	LP 3008	Aspartate, glycine, lysine; mannitol		Althabegoiti et al. (2008)

Note that information may conflict between reports.

∗ Results are disputed. See Compton et al. (2020).

## Attractants Classes of Model Rhizobia

When comparing results from different behavioral chemotaxis assays, one should bear in mind that attractant profiles of different species and strains will vary. Growth conditions (such as variations in media, temperature, aeration, and growth phase) can cause variances in receptor expression, altering the sensory capability of the bacterium (Lopez-Farfan et al., 2017). In addition, the literature is biased because some compounds have been tested more frequently than others and not to the same extent in different rhizobial species. However, it is naive to expect every source to standardize the array of compounds tested since most studies have a focus on a particular compound or component of plant exudates. As it stands, comparative analyses are restricted.

### Amino Acids

One of the most frequently tested compounds is amino acids because they are ubiquitous in exudates (Moe, 2013). *Rhizobium leguminosarum* biovar *viciae* is similarly attracted to all amino acids; only glutamate and proline stand out as stronger attractants (Gaworzewska and Carlile, 1982; Armitage et al., 1988). In contrast, *B. japonicum* was weakly attracted to non-acidic amino acids, while aspartate and glutamate were potent attractants (Barbour et al., 1991; Chuiko et al., 2002; Althabegoiti et al., 2008). *S. meliloti* senses all amino acids as attractants. Two groups presented aspartate or leucine as the strongest (Burg et al., 1982; Götz et al., 1982, respectively). However, these findings are in contrast with more recent reports that described the molecular mechanism for amino acid sensing, showing that arginine, phenylalanine, proline, and tryptophan are the strongest attractants of this class (Webb et al., 2017b). The chemoreceptor McpU directly binds all amino acids except for glutamate and aspartate, which do not serve as chemoattractants for *S. meliloti* RU11/001 (Webb et al., 2014, 2017b). The chemical nature of the R-group appears to be important for the bias of different bacterial species toward acidic or non-acidic amino acids.

### Carboxylates

Carboxylates, also referred to as organic acids, are carbon sources commonly found in plant exudates, the rhizosphere, and bulk soil. Citrate, malate, and succinate are attractants for *R. leguminosarum*, but are generally weaker than the amino acids (Gaworzewska and Carlile, 1982; Armitage et al., 1988). In *B. japonicum*, malonate and succinate elicit a strong attractant response, equivalent to aspartate and glutamate. In addition, other 4-carbon carboxylates are also attractants, although citrate is not (Barbour et al., 1991; Kape et al., 1991). *Sinorhizobium meliloti* has a dedicated 2–3 carbon monocarboxylate sensor, McpV, and a sensor for small dicarboxylates, McpT (Baaziz et al., in review; Compton et al., 2018). The mono- and di-carboxylates are weak attractants compared to the amino acids (Webb et al., 2014).

### Flavonoids

Flavonoids belong to a plant-borne compound group that are of interest because they induce the expression of symbiotic genes in their cognate rhizobia (Abdel-Lateif et al., 2012). Although apigenin and luteolin do not induce *nod* gene expression in *R. leguminosarum*, they are attractants in this organism. In contrast, naringenin is a *nod* gene inducer but not an attractant (Aguilar et al., 1988). Two reports on *B. japonicum* chemotaxis showed that all flavonoids tested have no attractant function (Barbour et al., 1991; Kape et al., 1991). Studies of *S. meliloti* chemotaxis reported that the *nod* gene inducers dihydroxyflavone and luteolin are attractants, but that responses were very low. The studies did not test any other compounds for comparison and, therefore, lack context (Caetanoanollés et al., 1988; Dharmatilake and Bauer, 1992). The conclusion that flavonoids are attractants was not replicated and recently disputed (Compton et al., 2020). Taken together, the evidence for flavonoid chemotaxis as a general phenomenon in rhizobia is debatable.

### Phenolics

The phenolics comprise a class of compounds that include phenylpropanoid derivatives of aromatic acids, which are common plant metabolites and precursors to flavonoids. *Bradyrhizobium japonicum* senses several phenylpropanoid acids and coniferyl alcohol as attractants, but not chlorogenic acid (Kape et al., 1991). Benzoic alcohols are attractants for *R. leguminosarum* with acetosyringone being one of the strongest attractants reported, while umbelliferone and vanillyl alcohol are weak to moderate attractants (Aguilar et al., 1988).

### Saccharides

Saccharides or sugars and their subclasses such as sugar alcohols and sugar acids are some of the most readily available carbon sources (Geddes and Oresnik, 2014). Common energy sources like gluconate and glucose as well as structural components of pectins such as arabinose and xylose are logical candidates to serve as attractants for rhizobia. Multiple reports investigated the taxis of *R. leguminosarum* to sugars, but these gave conflicting results. Glucose, maltose, ribose, and sucrose were reported as attractants in certain studies, but the evidence in other references suggests that they are not (Bowra and Dilworth, 1981; Gaworzewska and Carlile, 1982; Aguilar et al., 1988; Armitage et al., 1988). However, arabinose and xylose were identified as attractants in all studies, although they were not necessarily the strongest chemoattractants (Bowra and Dilworth, 1981; Gaworzewska and Carlile, 1982; Aguilar et al., 1988). Information on *B. japonicum* and *S. meliloti* taxis to sugars is sparse. Mannitol, a sugar alcohol, is the only member of this class that was presented as an attractant for *B. japonicum*, while common sugars such as arabinose and glucose do not appear to serve as attractants (Barbour et al., 1991; Chuiko et al., 2002). In *S. meliloti*, gluconate and sucrose were reported to be the best attractants among the sugars over arabinose, fructose, and glucose (Malek, 1989). Currently, it is difficult to make conclusions on the prevalence of sugars as chemoattractants in rhizobia. Most compounds were only tested in a single study and at one concentration (Table 1). Sugars are a large, complex class because of the numerous structural and stereoisomers, which further complicates analysis. Currently, there is too little information to make a clear statement about the role of sugars as attractants.

## Preferred Attractants for Rhizobia

A prominent aim in chemotaxis research is identifying dominant or preferred attractants for a given organism because it is indicative of its role in the ecosystem. The clearest body of information available for this conclusion comes from work done on *S. meliloti* strain RU11/001. All studies examining different classes of chemoattractants included comparative experiments with the chemoattractant proline and used similar techniques – thus creating a standard for comparison. This body of work allows the conclusion that amino acids are of similar attractant strength to quaternary ammonium compounds (QACs) such as betaines or choline (another class of attractants that was only recently recognized), and 5- to 10-fold stronger attractants than carboxylates (Webb et al., 2014, 2017a,b; Compton et al., 2018).

Work on the chemosensing of *B. japonicum*, albeit from a single study, revealed that glutamate, malonate, and succinate are the most potent attractants. It is noteworthy that glutamate was used as a nitrogen source in the growth medium, raising the possibility that chemotaxis responses are inducible in this organism (Barbour et al., 1991).

Acetosyringone is the strongest attractant reported for *R. leguminosarum*, followed by the flavonoids apigenin and luteolin. The evidence for rhizobial chemotaxis to flavonoids is strongest in *R. leguminosarum*, even though the bacterium only appears to be attracted to non-*nod* gene inducing flavonoid species (Aguilar et al., 1988). Arabinose and xylose are not the strongest attractants but have been consistently reported to attract multiple strains of *R. leguminosarum* (Bowra and Dilworth, 1981; Gaworzewska and Carlile, 1982; Aguilar et al., 1988).

## Where are Attractants Found?

### Seed Exudates

The compounds released from germinating seeds are a great source of attractants for rhizobia, readily available for study, and consistent between samples. When seeds are formed, the seed coat is impregnated with numerous chemicals from the mother plant (Radchuk and Borisjuk, 2014). Upon imbibition, these chemicals are leeched out as an exudate into the surrounding medium forming what is called the spermosphere (Nelson, 2004). Amino acids and carboxylates are some of the most sampled constituents of seed exudates and these compounds have the clearest link to known bacterial chemosensory systems (Götz et al., 1982; Rozan et al., 2001; Kuo et al., 2004; Kamilova et al., 2006; Lambers et al., 2013; Webb et al., 2016). However, there is great chemical diversity in exudates that include QACs such as betaines, saccharides, fatty acids, phenolics, and alcohols (Silva et al., 2013; Schiltz et al., 2015; Webb et al., 2017a; Mildaziene et al., 2020; Zuluaga et al., 2020). The gradients these chemicals form serve to recruit microorganisms to the surface of the nascent plant.

### Root Exudates

Exudates from roots share the chemical diversity of seed exudates, but their specific composition will vary according to biotic and abiotic factors and the state of the plant. Changes in exudate profiles are strongly driven by environmental factors (Herz et al., 2018; Dietz et al., 2019). Conditions lacking mineral nutrients induce the release of organic acids to solubilize phosphorus and chelate iron (Shen et al., 2005; Preece and Penuelas, 2020; Vives-Peris et al., 2020). Young or immature plants tend to release more sugars, while the exudates of older plants are biased toward amino acids and phenolics (Chaparro et al., 2013). In another example, a biocontrol organism induces the release of a plant metabolite that is toxic to a pathogen, but not to the biocontrol agent (Wang et al., 2019). In fact, and at the risk of overgeneralizing, it appears that nearly any environmental or physiological perturbation influences the composition or quantity of root exudates (Canarini et al., 2019; Vives-Peris et al., 2020). It would follow that chemotactic recruitment of soil bacteria is, in many cases, a byproduct of plant activity. In support of this, the saprotrophic soil bacterium *Pseudomonas putida* possesses a chemoreceptor that preferentially senses citrate-magnesium complex compared to free citrate, possibly sourced from efforts of a plant to acquire magnesium or other chelated cations (Martin-Mora et al., 2016). However, plants are not indifferent to their rhizosphere neighbors. Recruitment of soil symbionts is a definite priority for plants because of the benefits their microbiota provide. Furthermore, stressed plants release additional carbon compounds to recruit symbionts and other beneficial bacteria in times of need (Ames and Bergman, 1981; Gulash et al., 1984; Badri and Vivanco, 2009; Canarini et al., 2019; O’Neal et al., 2020; Vives-Peris et al., 2020).

### Other Attractant Sources

The vast majority of research focuses on rhizobial activities in the rhizosphere and nodule (Poole et al., 2018). Few studies examine rhizobial survival in the bulk soil. This is nonetheless an important topic, because effective inoculants and symbionts must persist in the soil to infect the next generation of hosts (Pinochet et al., 1993; Hirsch and Spokes, 1994; Da and Deng, 2003). In the absence of hosts, rhizobia can subsist on soil organic carbon, an amalgam containing energy and nutrient sources from detritus and decaying matter, which take the form of aromatics, alkyl compounds, carboxylates, and nitrogenous molecules such as proteins (Beyer et al., 1995; Chiu et al., 2002; Spielvogel et al., 2004). Rhizobia are adapted to saprophytic lifestyles, and chemotaxis to the carbon and nitrogen sources available is critical to survival in the bulk soil (Kennedy and Lawless, 1985; Turnbull et al., 2001; diCenzo et al., 2016; Poole et al., 2018).

Attractant signals need not be chemical compounds. Chemoreceptors can sense oxygen gradients or the cellular redox state as an indicator of the surrounding milieu, a strategy termed energy taxis. In the nitrogen fixing *Azospirilum brasilense*, metabolism mediates and is necessary for chemotaxis to attractants. This behavior is also critical to the colonization of host plants (Alexandre et al., 2000; Greer-Phillips et al., 2004). Utilizing a generalized strategy such as energy taxis allows a bacterium to seek areas suitable to its needs, regardless of the identity of the attractant source.

## Conclusion and Future Directions

**Figure 1:**
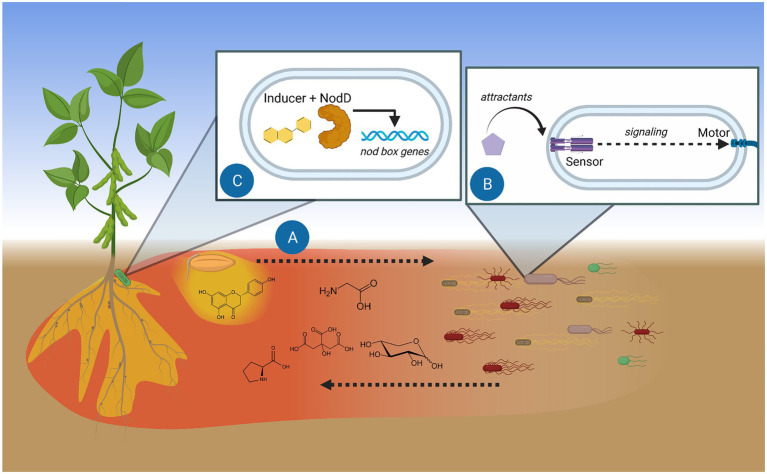
Model of chemotaxis function in the initiation of rhizobium-legume symbiosis. **(A)** Root and seed exudates diffuse and create chemical trails. Gradients of soluble exudates (red) travel furthest and act as chemoattractants. Hydrophobic exudate components (yellow) stay closer to the source. **(B)** Specific exuded compounds are detected by bacterial methyl-accepting chemotaxis proteins (MCPs), the dedicated sensors of attractants. Signaling between the chemosensory system and the motor guides the bacterium toward the attractant source. **(C)** Perception of plant flavonoids by rhizobial NodD initiates transcription of genes involved in infecting the proper host. Figure created with BioRender.

Chemotaxis is a major survival strategy for bacteria and greatly aids in seeking hosts (Raina et al., 2019). The rhizobia sense a wide array of chemical cues that are concomitantly found in the exudates of respective host plants but differ in their physiological role (**Figure 1**). Many of these attractants are primary metabolites and do not signify the identity of the source plant. A single chemical that is specific to a host would be difficult to pinpoint. Among semi polar metabolites, very few compounds are exclusively found in a particular plant species (Dietz et al., 2019). At best, specific metabolites might be characteristic of a genus or family (Rosenthal and Nkomo, 2000; Kidd et al., 2018). Rhizobia, therefore, have numerous chemoreceptors that may detect any of the thousands of compounds found in plant exudates, although in some cases, only a few chemoreceptors have any impact on host colonization (Feng et al., 2019). As each rhizobial species or strain analyzed seems to have different preferred attractants, the question remains how these preferences are formed. Since chemotactic preferences must be shaped by selection, perhaps each rhizobium specializes in obtaining particular types of nutrients. In effect, this is akin to a broad survival strategy that expands upon the phenomenon of catabolite repression, where a specific carbon source takes priority in the organism’s metabolism (Georgi and Ettinger, 1941; Bruckner and Titgemeyer, 2002; Iyer et al., 2016).

Every species or strain of rhizobia harbors numerous chemoreceptors that bestow perception of a particular signal. The number of MCPs per rhizobium varies greatly from less than 10 in *Sinorhizobium* and *Ensifer* spp. to over 30 in *R. leguminosarum* and *B. japonicum* isolates (for more information on chemoreceptor distributions, see Scharf et al., 2016). Currently, only four MCPs in *S. meliloti* have been assigned functions (Baaziz et al., in review; Webb et al., 2014, 2017b; Compton et al., 2018). The remainder of our knowledge on rhizobial chemotaxis is limited to dated behavioral studies. A complete understanding of rhizobium chemotaxis is an important goal for the following reasons: (1) total knowledge of a rhizobium’s chemotaxis system permits the modeling of its behavior in the environment and the prediction of its performance in host nodulation based on its exudate composition (Edgington and Tindall, 2018); (2) inoculant strains can be optimized to more efficiently nodulate crops, outcompete symbiotically inefficient native strains, and better survive in the soil, which increases the longevity of their application; and (3) chemoreceptors are often conserved among plant and animal pathogens, making information on MCPs translational to numerous fields (Brewster et al., 2016; Compton et al., 2018).

The way forward is, therefore, to increase the output of studies on MCP function that address the following goals: (1) identification of attractant classes and their relative strengths; (2) determination of sensors responsible for detecting attractants and the molecular mechanism of sensing; and (3) evaluation of the purpose of attractants in the survival or symbiosis of rhizobia. These aims can be easily initiated by revisiting known attractants and identifying their respective sensors. Two approaches are effective in characterizing MCP-ligand relationships. The first approach involves defining an attractant class and identifying a corresponding sensor, as exemplified by McpX in *S. meliloti* and the QACs (Webb et al., 2017a). The second approach identifies the ligand profile of an MCP using high-throughput screens followed by behavioral assays (McKellar et al., 2015). These pursuits will eventually illuminate each rhizobium’s attractome, the total range of compounds sensed through its chemotaxis systems. These findings will be a great boon to the economical, technical, and ecological challenges facing modern agriculture.

## Author Contributions

KC and BS contributed to conception of the study. KC wrote the first draft of the manuscript. Both the authors contributed to manuscript revision, read, and approved the submitted version.

### Conflict of Interest

The authors declare that the research was conducted in the absence of any commercial or financial relationships that could be construed as a potential conflict of interest.
